# Retinocytoma Undergoing Retinoblastoma Transformation in an Adult Patient

**DOI:** 10.1155/2023/8127245

**Published:** 2023-07-24

**Authors:** J. Navaratnam, R. Faber, N. Eide, M. Lund-Iversen, Ø. Garred, F. L. Munier

**Affiliations:** ^1^Department of Ophthalmology, Oslo University Hospital, Norway; ^2^Department of Pathology, Oslo University Hospital, Norway; ^3^Jules-Gonin Eye Hospital, Switzerland

## Abstract

We report a 46-year-old male patient with retinocytoma who presented at the age of 31 asymptomatically. An intraocular retinal mass was incidentally found in his right eye, when he underwent ophthalmological assessment for refractive surgery. This tumor consisted of a calcified sessile basis partially covered by a pedunculated salmon-pink growth. Initially, the tumor was diagnosed as a retinocytoma with clinical suspicion of malignant transformation into retinoblastoma and treated by four sessions of laser photocoagulation. Six and a half years later, the tumor relapsed, and he was treated with a Ruthenium plaque. Following brachytherapy, he had two episodes of right-sided vitreous hemorrhage that spontaneously cleared up, and the remaining finding in the vitreous cavity was interpreted as asteroid hyalosis. He underwent vitrectomy about five years following brachytherapy. The analysis of the vitreous material revealed the presence of inactive vitreous seeds composed of small round blue cells, compatible with a type 2 regression.

## 1. Introduction

Retinocytoma is a rare benign tumor of well-differentiated, mature retinal cells that very seldom undergo malignant transformation into retinoblastoma. It was first described by Gallie et al. as a retinoma with the characteristic clinical features that included a grey, translucent retinal mass, calcification, and hyperplastic retinal epithelium or chorioretinal scar [1]. Seeding may be seen in the vitreous cavity with calcifications [2]. Retinocytoma has been observed clinically between 1.8% and 3.2% of retinoblastoma cases [3, 4]. Histological findings of retinocytoma include eosinophilic cytoplasm, well-differentiated prominent photoreceptors (fleurettes), calcification, and nonproliferative cells [5]. Retinoblastoma demonstrates mitosis, necrosis, nuclear molding, infiltrating growth, and Homer Wright and Flexner-Wintersteiner rosettes [6]. Management of retinocytoma is mainly observation, and treatment is indicated if malignant transformation to retinoblastoma is suspected.

## 2. Case Presentation

**Figure 1:**
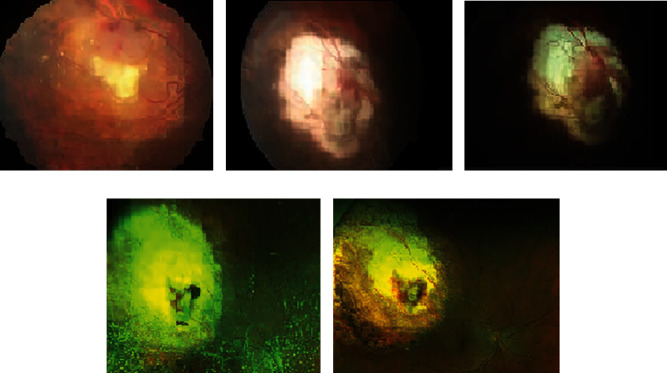
(a) The fundus image was taken at the patient's first presentation for evaluation of the tumor (Zeiss FF 450+; Carl Zeiss AG, Oberkochen, Germany). Fundus imaging revealed a tumor located in the superotemporal quadrant of his right eye. The upper part of the tumor appeared salmon-pink, and the lower part was calcified. Some asteroid hyalosis was seen. (b) This fundus image was taken following three laser treatments (Zeiss FF 450+; Carl Zeiss AG, Oberkochen, Germany). (c) The fundus image taken one month prior to brachytherapy demonstrated relapse of the tumor (salmon-pink colour) (Zeiss FF 450+; Carl Zeiss AG, Oberkochen, Germany). (d) Fundus image following brachytherapy and about 2 years prior to vitrectomy showed a denser asteroid hyalosis (Optos 200Tx; Optos PLC, Dunfermline, Scotland, United Kingdom). (e) Fundus imaging captured 8 months following pars plana vitrectomy (Optos 200Tx; Optos PLC, Dunfermline, Scotland, United Kingdom).

**Figure 2:**
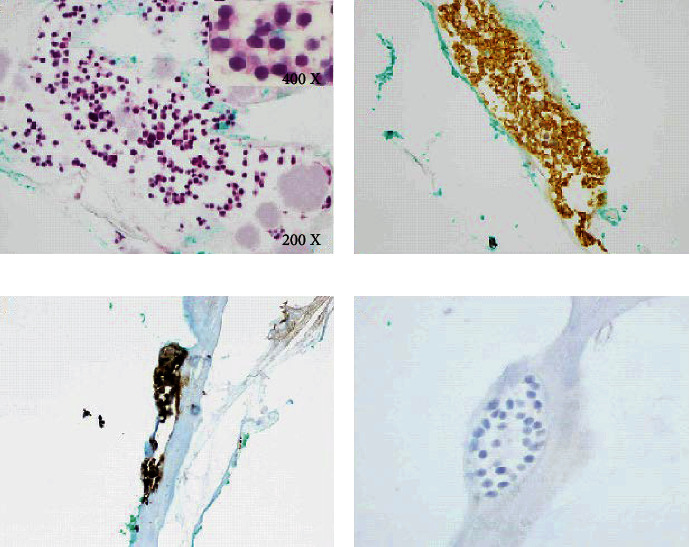
Vitreous samples (a–d): small groups of cells, marginally larger than lymphocytes, were seen microscopically (a; HE, 200X, and 400X in the upper corner). The cells had a high nuclear-cytoplasmic ratio, circular eccentric nuclei, and condensed chromatin. The cytoplasm was slightly eosinophilic, and mitotic activity was not present. In addition, there were several eosinophilic globules, consistent with asteroid hyalosis. The “tumor” cells expressed CD56 (b; 200X), synaptophysin (c; 200X), and neuron-specific enolase (NSE). They were negative for the lymphocytic marker CD45. The proliferation index assessed with Ki67 was less than 1% (d, 400X). Collectively, the findings were consistent with regressed vitreous seeds type 2.

The patient intended to undergo refractive surgery at the age of 31 due to professional requirements. The preoperative ophthalmological examination revealed a right-sided intraocular retinal mass, and he was referred for further examination and investigation. The family history was negative for ocular tumors. The best corrected visual acuity was 1.0 in both eyes, and the examination of the anterior segment of both eyes and the posterior segment of the left eye were within normal limits. Fundus examination of his right eye revealed an intraocular retinal mass located in the superotemporal quadrant (Figure 1(a)). The upper part of the tumor was salmon-pink in colour, measuring 3.4 × 4 × 2.7 mm, and the lower part appeared calcified with a size of 4.5 × 3.4 × 2.1 mm on B-scan ultrasonography. Retinal feeder vessels were observed on the surface of the tumor. The fluorescein angiography revealed pathological vessels early in the arterial phase with increasing leakage limited to the upper lesion. The radiological investigation with magnetic resonance imaging of his orbit and head revealed a right-sided contrast-enhanced intraocular tumor with a low T2 signal. The chest X-ray, ultrasound of the liver, and computed tomography of the abdomen revealed normal findings. The genetic investigation did not reveal constitutional mutation in the retinoblastoma (RB1) or von-Hippel Lindau (VHL) genes. The retinal mass was interpreted as a benign tumor or retinoma with respect to its calcified part, whereas its fish flesh counterpart was suspected to undergo malignant transformation and received four laser treatments to flatten the tumor. The height of the tumor shrank to 1.3 mm following laser treatments. However, 6.5 years later, the upper part of the lesion relapsed with a tumor thickness measuring 2.8 mm, requiring ruthenium brachytherapy (77 Gy to the tumor apex and 368 Gy to the sclera). Following brachytherapy, the patient experienced an increasing number of vitreous opacities, interpreted as asteroid hyalosis, and two episodes of vitreous hemorrhages. Five years following brachytherapy, when his BCVA was reduced to 0.6 due to increasing asteroid hyalosis (Figure 1(d)), he underwent pars plana vitrectomy without melphalan due to the complete regression of type 1 of the retinal tumor and type 2 of the vitreous seeding-like lesions. The cytology of the corpus vitreous sample revealed a nontumoral cellular component and asteroid hyalosis, a few macrophages (positive for CD68), and a few retinal cells without atypia (positive for CD56 and synaptophysin (Figures 2(b) and 2(c)) and negative for CD45). Unfortunately, there was not enough material left to perform immunostaining for CRX and pRB. The conclusion could not be made whether the retinal cells originated from normal retina or retinocytoma, but they were not malignant in nature and could be compatible with regression type 2 vitreous seeds. Two years following pars plana vitrectomy, his best corrected visual acuity was 1.6 bilaterally. Atrophy of the area treated with brachytherapy was seen (Figure 1(e)). The remaining mass was hyperechogenic, and the height of the lesion was size was reduced to 1.8 mm on B-scan ultrasonography.

## 3. Discussion

It is essential to differentiate retinocytoma from retinoblastoma since the latter is a malignant condition and requires treatment. Retinoblastoma is most likely to be diagnosed before the age of 5, while retinocytoma is commonly asymptomatic and more likely to be diagnosed in older children and adults. Retinoblastoma typically demonstrates growth within the first months and would most likely not have retinal pigment epithelial changes. Singh et al. reported a translucent retinal mass in 88%, calcifications in 63%, and retinal pigment epithelial changes in 54% of the retinocytoma cases. The presence of all three clinical features was found in 33% of retinocytoma patients [3]. Our patient's tumor had calcification inferiorly in contrast with fish flesh appearance superiorly.

Nonprogressive retinal tumor has been termed “spontaneous regression” of retinoblastoma, and affected patients may or may not carry a constitutional RB1 mutation [1]. The diagnosis of retinoblastoma can be made accurately by histological evaluation if the treatment necessities enucleation. Singh et al. reported malignant transformation of retinocytoma into retinoblastoma clinically characterized by an increase in the tumor size and vitreous seeding in 4% of their cases [3]. Differentiation between vitreous seeding from active retinoblastoma cells and asteroid hyalosis remains challenging due to similar appearances clinically and on ultrasonography. However, they can be distinguished easily since retinoblastoma seeds are spherical with obvious growth if left untreated by intravitreal chemotherapy. The vitreous sample obtained from the pars plana vitrectomy almost 5 years following brachytherapy revealed retinal cells without atypia, compatible with regression of type 2 of vitreous seeds [7].

Both retinocytoma and retinoblastoma carry biallelic mutations in the RB1 gene [8, 9]. Retinocytoma remains quiescent as long as tumor progression to retinoblastoma is not driven by additional hits. In rare cases, unilateral retinoblastoma can be caused by MYCN amplification in the context of a RB1-proficient background [10]. Our patient did not demonstrate a constitutional RB1 gene mutation.

Retinocytoma is diagnosed clinically and requires annual controls due to the risk of malignant transformation with an estimated lifelong probability of 5% [11].
